# Associations of childhood and current secondhand smoke exposure at home with current secondhand smoke exposure at work: A cross-sectional analysis of the Singapore multi-ethnic cohort

**DOI:** 10.18332/tid/174658

**Published:** 2023-12-15

**Authors:** Zilu Feng, Chuen Seng Tan, E-Shyong Tai, Jeong Kyu Lee

**Affiliations:** 1 Saw Swee Hock School of Public Health, National University of Singapore and National University Health System, Singapore, Singapore; 2 Yong Loo Lin School of Medicine, National University of Singapore, Singapore, Singapore; 3 Department of Health and Exercise Science, The University of Oklahoma, Norman, United States

**Keywords:** tobacco smoke pollution, family, working conditions, home environment

## Abstract

**INTRODUCTION:**

Relationships between secondhand smoke exposure (SHSE) in various temporal and physical settings are not fully studied despite its adverse impacts on human health, especially in multi-ethnic Asian populations. We investigated associations of childhood and current SHSE at home (SHSE_home_) with current SHSE at work (SHSE_work_) in Singapore and its relation to sources of daily smokers at home.

**METHODS:**

This cross-sectional study identified 925 healthy, never smoker working adults from the Singapore Multi-Ethnic Cohort (2004–2010). Firstly, the multiple logistic regression model estimated the adjusted odds ratios (AORs) of SHSE_home_. Subsequently, sources of daily smokers entered through an additional model building process using the former as a base.

**RESULTS:**

Current adults (AOR=2.05; 95% CI: 1.28–3.29) and childhood SHSE_home_ (AOR=1.43; 95% CI: 0.93–2.19) had a positive and no association with current SHSE_work_, respectively. These findings persisted when smoker identity-related variables entered the model: child (AOR=3.56; 95% CI: 1.19–10.64) for current daily smokers; father (AOR=2.30; 95% CI: 0.94–5.64) and sibling (AOR=2.97; 95% CI: 1.55–5.68) for childhood. Compared to no childhood SHSE_home_, only those living with their fathers and siblings who smoked daily at home had significantly higher odds of reporting current SHSE_work_ (AOR=3.70; 95% CI: 1.88–7.30).

**CONCLUSIONS:**

Current SHSE_home_ was a risk factor for current SHSE_work_, with risks elevated among those who smoke daily at home and living with their children. Childhood SHSE_home_ becomes a risk factor when daily household smokers include fathers and siblings. Deformalizing smoking could consider interpersonal dynamics of daily smokers at home with family members in different temporal settings, to reduce SHSE_work_.

## INTRODUCTION

Secondhand smoke exposure (SHSE) increases risks to many diseases, such as cardiovascular diseases and cancers in adults, and respiratory diseases and poor mental health outcomes in both children and adults^1^. Globally, SHSE causes an estimated 0.88 million deaths, of which 75% are non-smokers^2^. Hence, combating SHSE, particularly in workplaces and public spaces, is a key component of worldwide tobacco control policies. The World Health Organization (WHO) Framework Convention on Tobacco Control (FCTC) requires member states to enact comprehensive measures that protect the general population from SHSE at work (SHSE_work_) and public spaces^3^, such as legislating smoking bans in indoor workplaces, indoor public places, and public transport. This is necessary as workplaces without smoking restrictions increase the risk to SHSE, with some workplaces constituting higher risk than others^4,5^. Previous studies have reported that some groups of workers, such as males, ethnic minorities, younger workers, and those with lower education level^4,5^ have greater odds of SHSE_work_.

FCTC campaigns actively advocate for the adoption of smoke-free homes, as research shows children are at greater risk to SHSE than adults^6^, and more than 40% of youths globally are exposed to secondhand smoke (SHS) at home and have parents who smoke at home^7^. Risk of SHSE increases among children living with a smoker, and they have poorer health outcomes both during childhood and in the future as adults^1^. In particular, children living with a father or sibling who smoked at home is associated with earlier and/or greater odds of smoking initiation and having positive attitudes towards smoking and other tobacco-related health outcomes^8,9^. This suggests childhood SHSE and identity of the smoker at home may play a role in other future tobacco-related situations and outcomes, such as willingness to be exposed to SHS at work in adulthood. In addition, studies have shown that among people who are exposed to SHS at home, a vast majority are also exposed to SHS at work^10^. Overtime, SHSE at home (SHSE_home_) and at work (SHSE_work_) have decreased in tandem^11^. This suggests an association between SHSE at different physical settings.

To the best of our knowledge, the relationship between SHSE in different temporal and physical settings, especially within multi-ethnic Asian populations, are not fully understood. Singapore is a highly urbanized city-state in Asia with strict tobacco regulations, and had managed to lower smoking prevalence from almost 25% in the 1970s^12^ to 10% today^13^. Simultaneously, there is evidence that public perception and attitudes towards smoking has become less positive^14^, suggesting a shift towards social norms more hostile towards tobacco use, which is also observed in other developed economies^15,16^. As other Asian societies also become increasingly urban and cosmopolitan, Singapore’s multi-ethnic population provides an opportunity for this investigation. Studies from Western countries may be limited, given the need to contextualize the impact of culture and social norms on tobacco-related outcomes and behaviors in different societies^17^. We investigate the associations of childhood (age ≤18 years) and current SHSE_home_ with current SHSE_work_, among healthy adults who are never smokers, in Singapore, and whether these associations could be further explained by the sources of daily smokers at home.

## METHODS

### Study population

This cross-sectional study used information collected from Phase 1 of the Singapore Multi-Ethnic Cohort^18^ (MEC), a population-based study (see link for detailed information on the cohort: https://blog.nus.edu.sg/sphs/population-studies/multi-ethnic-cohort-phase-1-mec1/). In brief, recruitment of adults (age ≥21 years) from the three major ethnic groups (i.e. Chinese, Malay, Indian) in Singapore was conducted between 2004 and 2010, mainly from other existing cohort studies. Ethics approval for this sub-study was obtained from the Department Ethics Review Committee in Saw Swee Hock School of Public Health at National University of Singapore (SSHSPH-022).

Only never smoking, healthy and employed participants were included in this study. Participants were considered healthy if they responded ‘no’ to a series of questions regarding pre-existing chronic conditions in the survey (i.e. heart disease, stroke, high blood pressure, diabetes, high cholesterol, asthma, cancer, rheumatism, mental illness and other). Participants with missing information on any outcome, exposure and covariate variables were excluded from the analysis.

### Variables used in analysis

*Outcome*

SHSE_work_ was coded as 1 (or 0) when the response was ‘yes’ (or ‘no’) to the question: ‘Are you currently exposed to cigarette smoke at work on a daily basis?’.

*Exposures*

Childhood SHSE_home_ were coded as 1 (or 0) when the response was ‘yes’ (or ‘no’) to the questions: ‘From your birth to age 18 years, did anyone living in your home smoke at home on a daily basis for 6 months or longer?’ and ‘Does anyone who currently stays with you smoke on a daily basis?’. SHSE_home_ exposure characteristics in terms of sources of persons who smoked daily at home were available for selection by participants if they responded ‘yes’ to the previous questions. For childhood SHSE_home_, five exposure sources were available: 1) father, 2) mother, 3) sibling, 4) grandparent, and 5) other. For current SHSE_home_, responses were coded as 1 (or 0) when the response was ‘yes’ (or ‘no’) to the question: ‘Does anyone who currently stays with you smoke on a daily basis?’. Four exposure sources were identified: 1) spouse, 2) child, 3) parent/in-law, and 4) other. For each identity, a binary variable was generated indicating whether the response by the participant was ‘yes’.

*Covariates*

Covariates for the multiple logistic regression model were grouped into three broad categories: sociodemographics (age, gender, ethnicity, education level, marital status, and type of residence), lifestyle (ever drank alcohol, weekly physical activity level, and daily consumption of fresh fruits, vegetables, dairy products, poultry, red meat and seafood) and current health status proxied by clinical measurements [body mass index (BMI), mean arterial pressure (MAP), triglyceride level (TG), low-density lipoprotein cholesterol level (LDL), high-density lipoprotein cholesterol level (HDL) and hemoglobin A1c (HbA1c)]. All continuous variables were categorized into quartiles, except BMI which was categorized into four categories according to WHO’s recommendation for Asia-Pacific populations. These variables were chosen as they have been shown to be associated with tobacco-related health outcomes, including SHSE^19,20^.

### Statistical analysis

All analyses were conducted using R version 4.0.2. Frequencies and percentages were used to summarize categorical variables representing participants’ characteristics. Fisher’s exact test was used to assess the association between categorical variables and SHSE_work_.

A parsimonious multiple logistic regression model was developed using the forward stepwise approach to obtain adjusted odds ratios (AORs) of childhood and current SHSE_home_ on current SHSE_work_ (Model 1). All covariates were available for selection into the final model during the model building process while the variables in a base model, which included both temporal settings of SHSE_home_, were not allowed to exit during model building process. Covariates entered or exited at each step of the model building process based on whether they improved the model fit by utilizing the Akaike Information Criterion (AIC). Thereafter, Model 1 was the base model in the forward stepwise approach when investigating whether the associations of childhood and current SHSE_home_ with SHSE_work_ could be further explained by the sources of daily smokers at home (Model 2). All identity-related variables (i.e. all binary variables related to sources of daily smokers) were available for selection into the final model during the model building process (i.e. forward stepwise approach). Interaction between the childhood and current SHSE_home_ were assessed to determine whether the two effects of SHSE_home_ were independent. If these effects were not independent, all identity-related variables in Model 2 had their two-way interactions assessed regardless whether they were sources from childhood or current. Otherwise, identity-related variables from the same temporal setting in Model 2 had their two-way interactions assessed where applicable. For each AOR that was attributable to participants from the same SHSE_home_ profile (e.g. experienced childhood SHSE_home_ and lived with father and sibling(s) who smoked daily at home during childhood), it was decomposed into AORs that corresponded to SHSE_home_ characteristics (i.e. childhood and current SHSE_home_, and identity-related variables) in Model 2. To corroborate the computed AORs of subgroups, the categorical variable representing the subgroups would be included as a predictor in the model and adjusted for the same covariates as Model 2 (Model 3). See the Supplementary file for further elaboration on this decomposition.

## RESULTS

**Figure 1:**
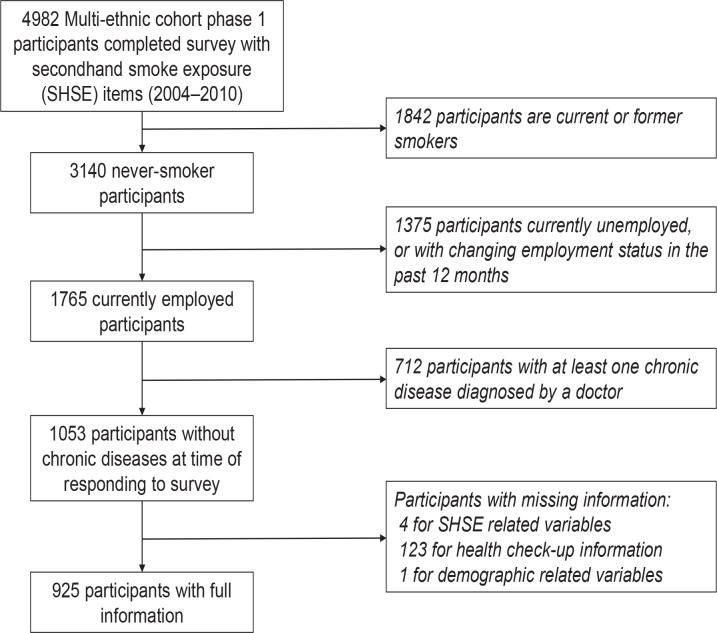
Study participants selection flow chart

Figure 1 is the flow chart of participants’ selection that included 925 never smokers, employed, and healthy participants with complete information used in this analysis. Table 1 presents the characteristics of the participants. Overall, the majority of participants were female (66.8%), of Chinese ethnicity (45.3%), had pre-university education (37.0%), lived in HDB 4-room flats (40.3%), and were married (68.1%). The average age of participants was 38.7 years (SD=10.5). In total, 144 (15.6%) were exposed to SHS at work, while 217 (23.5%) were currently exposed to SHS at home; and among them the two most common sources of SHSE_home_ were the spouse (47.5%) and parent/in-law (28.1%). Similarly, 483 participants (52.2%) were exposed to daily SHSE_home_ during childhood. Among those respondents, the two most common sources of exposure were the father (86.7%) and siblings (21.9%). Having at least one sibling who smoked daily at home during childhood was significantly associated with current SHSE_work_. For current SHSE_home_, having at least one parent or parent in-law and at least one other source who smoked daily at home was significantly associated with reporting current SHSE_work_. Participants who were younger, males, Indians, with pre-university education, never married, those who reported a lower consumption of fresh fruits, lower HDL and lower HbA1c, were more likely to report current SHSE_work_.

**Table 1:** Characteristics of participants (N=925)

*Characteristics of SHSE at home (SHSE_home_)*	*Current SHSE at work (SHSE_work_) on a daily basis*			
	*Total (N=925)*	*Yes (N=144; 15.6%)*	*No (N=781; 84.4%)*	*p^a^*
	*n (%)*	*n (%)*	*n (%)*	
**SHSE_home_ on a daily basis**				
**Temporal setting: Childhood** (age ≤18 years)				0.084
No	442 (47.8)	59 (13.3)	383 (86.7)	
Yes	483 (52.2)	85 (17.6)	398 (82.4)	
**Temporal setting: Current**				0.001
No	708 (76.5)	94 (13.3)	614 (86.7)	
Yes	217 (23.5)	50 (23.0)	167 (77.0)	
**Identities of daily smokers at home**				
**Temporal setting: Childhood** (age ≤18 years)				
Father	419 (45.3)	76 (18.1)	343 (81.9)	0.056
Mother	21 (2.3)	2 (9.5)	19 (90.5)	0.759
Sibling	106 (11.5)	26 (24.5)	80 (75.5)	0.010
Grandparent	16 (1.7)	2 (12.5)	14 (87.5)	1.000
Other	19 (2.1)	3 (15.8)	16 (84.2)	1.000
**Temporal setting: Current**				
Spouse	103 (11.1)	15 (14.6)	88 (85.4)	0.886
Child	27 (2.9)	8 (29.6)	19 (70.4)	0.055
Parent/in-law	61 (6.6)	17 (27.9)	44 (72.1)	0.010
Other	51 (5.5)	16 (31.4)	35 (68.6)	0.004
**Sociodemographics**				
**Age** (years)				<0.001
Q1: <30	216 (23.4)	54 (25.0)	162 (75.0)	
Q2: 30 to <39	236 (25.5)	37 (15.7)	199 (84.3)	
Q3: 39 to <46	225 (24.3)	28 (12.4)	197 (87.6)	
Q4: ≥46	248 (26.8)	25 (10.1)	223 (89.9)	
**Gender**				<0.001
Female	618 (66.8)	58 (9.4)	560 (90.6)	
Male	307 (33.2)	86 (28.0)	221 (72.0)	
**Ethnicity**				0.001
Chinese	419 (45.3)	45 (10.7)	374 (89.3)	
Malay	222 (24.0)	42 (18.9)	180 (81.1)	
Indian	284 (30.7)	57 (20.1)	227 (79.9)	
**Education level**				<0.001
Primary and lower	157 (17.0)	19 (12.1)	138 (87.9)	
Secondary	251 (27.1)	28 (11.2)	223 (88.8)	
Pre-university	342 (37.0)	79 (23.1)	263 (76.9)	
University and higher	175 (18.9)	18 (10.3)	157 (89.7)	
**Housing type^b^**				0.481
HDB 1–2 rooms	26 (2.8)	4 (15.4)	22 (84.6)	
HDB 3 rooms	177 (19.1)	34 (19.2)	143 (80.8)	
HDB 4 rooms	373 (40.3)	57 (15.3)	316 (84.7)	
HDB 5 rooms/executive flat/condo/landed/other	349 (37.7)	49 (14.0)	300 (86.0)	
**Marital status**				0.007
Married	630 (68.1)	82 (13.0)	548 (87.0)	
Never married	245 (26.5)	52 (21.2)	193 (78.8)	
Widowed/divorced/separated	50 (5.4)	10 (20.0)	40 (80.0)	
**Consumption habits**				
**Alcoholic drink**				0.103
No	461 (49.8)	81 (17.6)	380 (82.4)	
Yes	464 (50.2)	63 (13.6)	401 (86.4)	
**Fresh fruits** (g/day)				<0.001
Q1: <100.8	231 (25.0)	41 (17.7)	190 (82.3)	
Q2: 100.8 to <188.0	232 (25.1)	51 (22.0)	181 (78.0)	
Q3: 188.0 to <303.8	230 (24.9)	18 (7.8)	212 (92.2)	
Q4: ≥303.8	232 (25.1)	34 (14.7)	198 (85.3)	
**Vegetables** (g/day)				0.129
Q1: <100.2	231 (25.0)	30 (13.0)	201 (87.0)	
Q2: 100.2 to <152.4	231 (25.0)	42 (18.2)	189 (81.8)	
Q3: 152.4 to <220.1	231 (25.0)	43 (18.6)	188 (81.4)	
Q4: ≥220.1	232 (25.1)	29 (12.5)	203 (87.5)	
**Dairy products** (g/day)				0.241
Q1: <75.9	232 (25.1)	27 (11.6)	205 (88.4)	
Q2: 75.9 to <151.3	231 (25.0)	40 (17.3)	191 (82.7)	
Q3: 151.3 to <287.1	230 (24.9)	36 (15.7)	194 (84.3)	
Q4: ≥287.1	232 (25.1)	41 (17.7)	191 (82.3)	
**Poultry** (g/day)				0.244
Q1: <23.0	232 (25.1)	30 (12.9)	202 (87.1)	
Q2: 23.0 to <40.8	231 (25.0)	40 (17.3)	191 (82.7)	
Q3: 40.8 to <70.5	231 (25.0)	31 (13.4)	200 (86.6)	
Q4: ≥70.5	231 (25.0)	43 (18.6)	188 (81.4)	
**Red meat** (g/day)				0.350
Q1: <11.6	231 (25.0)	35 (15.2)	196 (84.8)	
Q2: 11.6 to <24.1	231 (25.0)	44 (19.0)	187 (81.0)	
Q3: 24.1 to <53.6	231 (25.0)	30 (13.0)	201 (87.0)	
Q4: ≥53.6	232 (25.1)	35 (15.1)	197 (84.9)	
**Seafood** (g/day)				0.819
Q1: <32.5	231 (25.0)	37 (16.0)	194 (84.0)	
Q2: 32.5 to <55.2	231 (25.0)	36 (15.6)	195 (84.4)	
Q3: 55.2 to <83.8	231 (25.0)	39 (16.9)	192 (83.1)	
Q4: ≥83.8	232 (25.1)	32 (13.8)	200 (86.2)	
**Weekly physical activity level** (met-hr/week)				0.215
Q1: <63.5	231 (25.0)	35 (15.2)	196 (84.8)	
Q2: 63.5 to <99.8	231 (25.0)	27 (11.7)	204 (88.3)	
Q3: 99.8 to <136.6	231 (25.0)	40 (17.3)	191 (82.7)	
Q4: ≥136.6	232 (25.1)	42 (18.1)	190 (81.9)	
**Current health status**				
**Body mass index** (kg/m^2^)				0.417
Underweight: <18.5	42 (4.5)	3 (7.1)	39 (92.9)	
Normal: 18.5 to <23.0	332 (35.9)	50 (15.1)	282 (84.9)	
Overweight: 23.0 to <27.5	314 (33.9)	50 (15.9)	264 (84.1)	
Obese: ≥27.5	237 (25.6)	41 (17.3)	196 (82.7)	
**Triglyceride level** (mmol/L)				0.991
Q1: <0.62	222 (24.0)	34 (15.3)	188 (84.7)	
Q2: 0.62 to <0.87	239 (25.8)	36 (15.1)	203 (84.9)	
Q3: 0.87 to <1.30	231 (25.0)	37 (16.0)	194 (84.0)	
Q4: ≥1.30	233 (25.2)	37 (15.9)	196 (84.1)	
**Low-density lipoproteins** (mmol/L)				0.068
Q1: <2.72	229 (24.8)	31 (13.5)	198 (86.5)	
Q2: 2.72 to <3.25	232 (25.1)	27 (11.6)	205 (88.4)	
Q3: 3.25 to <3.87	232 (25.1)	46 (19.8)	186 (80.2)	
Q4: ≥3.87	232 (25.1)	40 (17.2)	192 (82.8)	
**High-density lipoproteins** (mmol/L)				<0.001
Q1: <1.00	221 (23.9)	46 (20.8)	175 (79.2)	
Q2: 1.00 to <1.24	236 (25.5)	41 (17.4)	195 (82.6)	
Q3: 1.24 to <1.48	229 (24.8)	39 (17.0)	190 (83)	
Q4: ≥1.48	239 (25.8)	18 (7.5)	221 (92.5)	
**Hemoglobin A1c** (%)				0.009
Q1: <5.5	205 (22.2)	39 (19.0)	166 (81.0)	
Q2: 5.5 to <5.7	223 (24.1)	46 (20.6)	177 (79.4)	
Q3: 5.7 to <5.9	241 (26.1)	27 (11.2)	214 (88.8)	
Q4: ≥5.9	256 (27.7)	32 (12.5)	224 (87.5)	
**Mean arterial pressure** (mmHg)				0.141
Q1: <75.3	230 (24.9)	30 (13.0)	200 (87.0)	
Q2: 75.3 to <82.3	218 (23.6)	39 (17.9)	179 (82.1)	
Q3: 82.3 to <90.3	244 (26.4)	31 (12.7)	213 (87.3)	
Q4: ≥90.3	233 (25.2)	44 (18.9)	189 (81.1)	

a Fisher’s exact test.

b Almost 80% of Singapore population lives in public housing known as HDB (Housing Development Board) flats, which are rented or bought from the government, with eligibility and the amount subsidised determined by household income, and size of apartment sought.

Q1: quartile 1. Q2: quartile 2. Q3: quartile 3. Q4: quartile 4. SHSE: secondhand smoke exposure.

**Figure 2:**
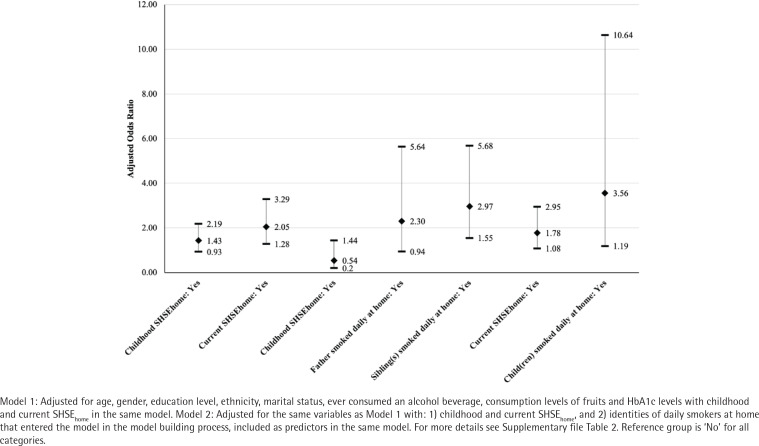
Associations between characteristics of secondhand smoke exposure (SHSE) at home (SHSE_home_) and current SHSE at work (SHSE_work_)

Figure 2 presents the associations between characteristics of SHSE_home_ and current SHSE_work_, after adjusting for the covariates that enter the model through model building (i.e. age, gender, education level, ethnicity, marital status, ever consumed an alcohol beverage, consumption levels of fruits, and HbA1c levels). Association for childhood SHSE_home_ (Model 1: AOR=1.43; 95% CI: 0.93–2.19) and current SHSE_home_ (Model 1: AOR=2.05; 95% CI: 1.28–3.29) with current SHSE_work_ were both positive, but only the latter was statistically significant. When sources of daily smokers at home were entered into Model 1 through model building, the association for childhood SHSE_home_ remained non-significant (Model 2: AOR=0.54; 95% CI: 0.20–1.44) while the association for current SHSE_home_ (Model 2: AOR=1.78; 95% CI: 1.08–2.95) remained significant and positive.

Two out of the five sources of exposure to SHS during childhood were then entered into the model (i.e. father and sibling). Specifically, living with a father (Model 2: AOR=2.30; 95% CI: 0.94–5.64) and at least one sibling (Model 2: AOR=2.97; 95% CI: 1.55–5.68) who smoked daily at home during childhood was positively associated with current SHSE_work_, where the latter was significant. Only one out of the four sources from current enter the model (i.e. child). Specifically, living with at least one child who currently smokes daily at home was associated with current SHSE_work_ (Model 2: AOR=3.56; 95% CI: 1.19–10.64). We did not find a significant interaction between childhood and current SHSE_home_ in Model 1 (p=0.914) and Model 2 (p=0.755). Similarly, interactions between the two sources of exposure from childhood that enter the model were not significant (p=0.626).

Based on the sources that enter Model 2, we classified participants into subgroups that contain SHSE_home_ profiles with the same adjusted odds ratio. Since the interaction analyses suggest the associations of childhood and current SHSE_home_ are independent, Table 2 presents the AORs of subgroups at each temporal setting with the reference subgroup corresponding to no SHSE_home_ in the same temporal setting. The AOR between the subgroup with no childhood SHSE_home_ and each of the subgroups experiencing childhood SHSE_home_ were not significantly different except for the subgroup with combinations of SHS sources that include both father and sibling as daily smokers at home (Model 2: AOR=3.70; 95% CI: 1.88–7.30). This subgroup contains combinations, such as father and sibling only, and father and sibling with one or more of the following three sources of exposure: mother, grandparent, and other.

**Table 2:** Associations between subgroups of secondhand smoke exposure (SHSE) at home (SHSE_home_) profiles and current SHSE at work (SHSE_work_)

*Subgroups of SHSE_home_ profiles on a daily basis*		*Model 2*			*Model 3*		
	*n (%)*	*AOR*	*95% CI*	*p*	*AOR*	*95% CI*	*p*
***Temporal setting: Childhood*** (age ≤18 years)							
**No childhood SHSE_home_** (Ref.)	442 (47.8)	1			1		
**Experienced childhood SHSE_home_ and combinations of identities^a^**							
Excluded father and sibling^b^	28 (3.0)	0.54	0.20–1.44	0.220	0.70	0.18–2.81	0.618
Included father but excluded sibling^c^	349 (37.7)	1.25	0.78–1.99	0.348	1.23	0.77–1.97	0.383
Excluded father but included sibling^d^	36 (3.9)	1.61	0.65–3.97	0.301	1.42	0.50–4.06	0.511
Included both father and sibling^e^	70 (7.6)	3.70	1.88–7.30	<0.001	3.90	1.92–7.89	<0.001
***Temporal setting: Current***							
**No current SHSE_home_** (Ref.)	708 (76.5)	1			1		
**Experienced current SHSE_home_ and combinations of identities^a^**							
Excluded child^f^	190 (20.5)	1.78	1.08–2.95	0.024	1.78	1.07–2.94	0.025
Included child^g^	27 (2.9)	6.35	2.19–18.40	0.001	6.43	2.21–18.67	0.001

AOR: adjusted odds ratio. Model 2: Obtained AORs of subgroups by multiplying relevant AORs of SHSE_home_ characteristics from Model 2 (see Table 2). Model 3: Included two categorical variables representing the subgroups of childhood and current SHSE_home_ profiles as predictors in the model with adjustment for the same covariates as Model 2 (see footnotes in Table 2 for details of covariates).

a Identities were daily smokers at home during specific temporal settings.

b One or more of these identities: mother, grandparent and other.

c Father only, and father with one or more identities listed in footnote b.

d Sibling only, and sibling with one or more identities listed in footnote b.

e Father and sibling only, and father and sibling with one or more identities listed in footnote b.

f One or more of these identities: spouse, parent/in-law and other.

g Child only, and child with one or more identities listed in footnote f.

For current SHSE_home_, the subgroup experiencing current SHSE_home_ with combinations of sources that exclude children as the current daily smoker at home (i.e. combinations with one or more of these three sources: spouse, parent/in-law, and other) had significantly higher odds of reporting exposure than the subgroup with no current SHSE_home_ (Model 2: AOR=1.78; 95% CI: 1.08–2.95). When the combinations of sources include also children in the home, the odds further increased (Model 2: AOR=6.35; 95% CI: 2.19–18.40). Similar results were obtained when we represented these subgroups of SHSE_home_ profiles from two temporal settings as two categorical variables and include them as predictors into one model with adjustment for the same covariates as Model 2 (Model 3).

## DISCUSSION

We investigate associations of childhood SHSE_home_ and current SHSE_home_ with current SHSE_work_, among healthy and employed adults who were never smokers, in Singapore. Although it is only the association between current SHSE_home_ and SHSE_work_ that is significant and positive, the associations of childhood and current SHSE_home_ with SHSE_work_ are further explained by the sources of daily smokers at home. For childhood SHSE_home_, the two sources were the father and siblings. Although both sources increase the odds of current SHSE_work_, only exposure via siblings was significant. However, when compared to no childhood SHSE_home_, the odds were only significantly higher among exposed participants who lived with their fathers and siblings who smoked daily at home during their childhood. For current SHSE_home_, regardless of the identity of daily smokers at home, the odds among the exposed is significantly higher than the odds among the unexposed. But participants living with their children who are current daily smokers at home led to significantly elevated odds.

Our results highlight the need to consider sources of smokers at home to gain deeper insights into the impact of SHSE_home_ on current SHSE_work_, indicating the importance of familial relationships and dynamics. Considering only the occurrence of SHSE without factoring in the other underlying characteristics of SHSE could prematurely lead to false negative findings for childhood SHSE_home_. Existing studies found social relationships are closely linked with tobacco use in general^21^, and hence could potentially be linked with other tobacco-related issues, such as SHSE in different physical settings and throughout one’s life-course. Parents’, siblings’ and peers’ attitudes and behaviors play significant roles in shaping children’s and adolescents’ attitudes towards tobacco smoking^8,9^, and evidence supports that the roles played by these different social relations are independent. Similarly, our results suggest that although living with either father or sibling who smoked increase the odds of current SHSE_work_ independently, it is the sibling identity that may play a greater role.

When the sources of childhood daily smokers at home include both father and sibling, the odds of current SHSE_work_ is significantly higher than those with no childhood SHSE_home_. The smoking behavior of father and sibling(s) at home could have established social norms by signaling that smoking and exposing others to SHS is acceptable^16^. It has been posited that social norms are one of the important processes through which one’s environment can affect behaviours^22^. Within Asian households^23^, fathers are, generally, in a position of authority, and siblings could be viewed as both family members and peers to the individual^8,24^. Their collective behaviors at home could enhance the prominence of the normative behaviour^25^ of smoking in the presence of others. When such behaviors are more commonly observed, individuals could be more susceptible to other factors that also affect tobacco-related health behaviors.

Efforts to promote smoke-free homes by focusing on the household level have been proven to be effective in lowering SHSE in children^26^. Given the potential impact of various sources of smokers during childhood on tobacco-related behavior and health outcomes in adulthood, efforts to alleviate SHSE_home_ should take the family context into consideration, ensuring the involvement of family members who are daily smokers at home. This is particularly pertinent during one’s childhood, as children typically have limited power to change the behavior of adults they are living with.

In contrast to findings in childhood SHSE_home_, the association of current SHSE_home_ was positive and significant. This could suggest current norms in the home environment is spread to the workplace or vice-versa^27^, given that SHSE_home_ and SHSE_work_ are happening at the same period. The prevailing social and environmental norms are likely acting on both the home and workplace setting^28,29^, hence smoke-free policies have the potential to enhance changes in social norms surrounding tobacco-related behavior at the broader, community level^30^. When the current daily smokers at home include the child identity, the odds of current SHSE_work_ are elevated more than for the subgroups that include the other current sources only. Prior evidence has shown that parents of smokers tend to have non-negative attitudes towards smoking^31,32^.

Overall, this study implies that different smoker sources at different phases of an individual’s life may have an impact on accepting, and hence reporting SHSE_work_. We examined different sources at different temporal settings, reflecting the changing life-course of the participant. Although we can distinguish between the specific parent who smoked daily at home during childhood, we are unable to do so for current SHSE_home_, as they are grouped with the in-laws in the survey. The changing impact of family dynamics^33^ on SHSE_home_ as one ages could be further studied using qualitative methods.

### Limitations

Our study has limitations. As it is cross-sectional in nature, we cannot establish the causal relationship between current exposure to SHS at home and work. Use of self-reported SHSE is susceptible to social desirability bias and recall bias. Our data captured SHSE_home_ only from those living in the same household, hence we cannot account for other modes of SHSE_home_, such as drifting SHS from neighboring apartments. This would be pertinent in high-density apartment dwellings common in Singapore, which are at higher risk to SHSE_home_ due to drifting smoke from neighbours^34^. SHSE_home_ may have been exacerbated by the COVID-19 pandemic, as many were working and learning from their homes at the time of the study. Drifting SHS from neighboring apartments could be explored in future studies.

### Implications

Limitations notwithstanding, our results, taken together with existing studies, demonstrate the importance of reducing SHSE_home_ in both temporal settings. Specifically, public health policies should target creating smoke-free home environments for children, who are not in the position to influence the actions of their parents or older siblings^30^, thus lowering the likelihood of childhood SHSE_home_ and the children commencing smoking in the future^16^. Community level interventions, especially those focusing on shifting existing social norms on tobacco-related health behaviors, have been found to lead to household and individual level changes^30^. For example, implementing smoke-free regulations at the workplace is found to be associated with more homes also becoming smoke-free^11,35^. However, such efforts should consider local contexts^36^.

## CONCLUSIONS

In general, our study found positive associations between SHSE_home_ and SHSE_work_, with the sources of daily smokers at home playing an important role among working adults who are healthy, never smokers, in Singapore. Current SHSE_home_ is a risk factor for current SHSE_work_ and the subgroup with the highest risk consists of individuals who are currently living with their children who smoke daily at home. Childhood SHSE_home_ may have an impact on current SHSE_work_, particularly among individuals who lived with their fathers and siblings who smoked daily at home, during their childhood. These results provide additional evidence on the importance of living in a smoke-free home environment because it reduces exposure to SHS at work. Deformalizing smoking in the presence of others at home, in different temporal phases of an individual’s life-course, could lead to meaningful changes in the individual’s exposure to SHS at work.

## Data Availability

The data supporting this research are available from the following source: https://blog.nus.edu.sg/sphs
